# Artificial Intelligence, Machine Learning and Deep Learning in Ion Channel Bioinformatics

**DOI:** 10.3390/membranes11090672

**Published:** 2021-08-31

**Authors:** Md. Ashrafuzzaman

**Affiliations:** Department of Biochemistry, College of Science, King Saud University, Riyadh 11451, Saudi Arabia; mashrafuzzaman@ksu.edu.sa

**Keywords:** ion channel, bioinformatics, artificial intelligence, deep learning, machine learning, channel classification, mutation

## Abstract

Ion channels are linked to important cellular processes. For more than half a century, we have been learning various structural and functional aspects of ion channels using biological, physiological, biochemical, and biophysical principles and techniques. In recent days, bioinformaticians and biophysicists having the necessary expertise and interests in computer science techniques including versatile algorithms have started covering a multitude of physiological aspects including especially evolution, mutations, and genomics of functional channels and channel subunits. In these focused research areas, the use of artificial intelligence (AI), machine learning (ML), and deep learning (DL) algorithms and associated models have been found very popular. With the help of available articles and information, this review provide an introduction to this novel research trend. Ion channel understanding is usually made considering the structural and functional perspectives, gating mechanisms, transport properties, channel protein mutations, etc. Focused research on ion channels and related findings over many decades accumulated huge data which may be utilized in a specialized scientific manner to fast conclude pinpointed aspects of channels. AI, ML, and DL techniques and models may appear as helping tools. This review aims at explaining the ways we may use the bioinformatics techniques and thus draw a few lines across the avenue to let the ion channel features appear clearer.

## 1. Introduction

The use of artificial intelligence (AI) in bioinformatics and computational molecular biology research has been growing fast over the last two decades [1,2]. Bioinformatics methods attempt to model known biological structures and predict unknown ones. Versatile bioinformatics techniques are capable of storing the information processed in various biological and biophysical studies in the created databank, and calling and utilizing the information from the databank in pinpointing crucial molecular processes of an individual system or collective ones. The techniques thus help establish scientific links between various mechanisms and processes and produce concluding evidence that is otherwise often unattainable using conventional theoretical and experimental techniques. Besides, computational techniques are popularly found to model the biomolecular complexes in silico studies to mainly address their statics, dynamics, and energetics in an artificially constructed, yet mimicking the biological systems’ environment.

Although about just 2% of the protein structures that are experimentally identified are among the transmembrane proteins (many of which construct ion channels), genome studies suggest that these special proteins together make up about 30% of all of the coded proteins. While mapping the membrane proteome Almén and colleagues found 27% of the total human proteome to be α-helical transmembrane proteins [3]. Bioinformatics method enables modeling of the unknown structure of the proteins, predicts their functions, their transmembrane location, and their ligand binding potency. Current in silico modeling tools use various computational methods, which are capable of providing results that may mimic nearly the biologically relevant functionality. General understanding of genetics, the gene-based mutations, emergence of disease, etc., as well as information on even evolution that concern ion channel structures and functions including both normal and abnormal biological systems’ status quos may be addressed using bioinformatics techniques. A huge amount of data from all this research contain information about certain biological systems, processes or mechanisms. These data and information are stored at various locations and sites utilizing random methods. Pulling them with the use of valid scientific ways and processing towards constructing any meaningful conclusions are challenging tasks. AI techniques appear as helpful tools to deal (extract, process and analyze) with such kind of big biological research data [4]. Knowledge on computing models using AI, advanced analytics of data and various optimization approaches that are used in bioinformatics, bioengineering and biophysics research on designing drugs and related analysis, medical imaging data analysis, biologically inspired artificial learning and adaption for general analytics, etc. is very useful. This knowledge is often found applicable in understanding many specific aspects of ion channels.

### Association of AI, ML, and DL Techniques with Ion Channel Bioinformatics

DL is a subset of ML and ML is a subset of AI: AI(ML(DL))). For ML, machines are supposed to learn and adapt through experience; for AI, machines can smartly execute specified tasks. DL is basically concerned with specific algorithms that are inspired by the human brain structure and function, known commonly as artificial neural networks (ANNs). The opportunity of using AI techniques in system biology is enormous [5]. ML techniques appear as powerful tools with capability to extract information from any data sets which are massive in size and noisy in nature. A review has described approaches that are based on simultaneous use of the systems biology and the ML in order to access the gene and the protein druggability [6]. It also elaborated on the sources of data, algorithms, and performance of different methods. The mathematical and computational methodologies underlying DL models appear quite challenging for interdisciplinary scientists, who may consult a recent review for being familiar with the techniques [7]. This article has presented a review on introduction to DL approaches that include Convolutional Neural Networks (CNNs), Deep Feedforward Neural Networks (D-FFNN), Deep Belief Networks (DBNs), Autoencoders (AEs), Long Short-Term Memory (LSTM) networks; many (if not all) of which have already found applications in bioinformatics field dealing with biological structures and functions.

AI has long been found useful in bioinformatics, and computational molecular biology (e.g., especially in the field of DNA sequencing) [1]. The main use of AI in these fields is in understanding of the organisms’ evolution, and slow growth of complexity of working with data having errors. AI softwares and modeling help to search, make classification and mine versatile biological databases; and especially simulate biological, physiological, biochemical, and biophysical experiments with and without errors. AI techniques are now found generally useful to handle (process, understand and create conclusion on specific aspects) partially the human genome data with billions of basepairs (bps), the necessity of what was rigorously addressed in ref. [8].

In an ML paper on bioinformatics, two decades ago, Tan and Gilbert analyzed learning systems (7 individual ones) and methods (9 combined ones) using 4 data sets of biological systems, and provided a few crucial issues (which are still considered generally applicable) to follow while answering a few questions on choosing correct algorithm to best suit for a data set, possibility of having any combined method(s) which might be better than especially any singular approach, comparing the effectiveness of any particular algorithm over others, etc. [9]. Even about three decades ago, people used ML approaches for gene recognition [10].

ML techniques; the ANN and the support vector machine (SVM) have been recently found to help predict the secretory proteins that may not necessarily require the presence or absence of the N-terminal signaling peptides, which are commonly known as the classical and the non-classical secreted proteins [11]. Here the methods have been trained and tested on a dataset of 3321 secretory and 3654 non-secretory proteins of mammals have been used to train the methods here with the use of a technique consisting of five-fold cross-validations. ANN-based modules were developed for mainly predicting the secretory proteins where 33 physicochemical properties, with compositions of the amino acids and the dipeptide, were considered. Considerable accuracies (73.1%, 76.1%, and 77.1%, respectively), were achieved. SVM-based modules used 33 physicochemical properties, with the compositions of amino acid, and the dipeptide and found similar accuracies (77.4%, 79.4%, and 79.9%, respectively). Basic Local Alignment Search Tool, commonly known as BLAST and the Position-Specific Iterative BLAST (PSI-BLAST) modules got designed for the purpose of predicting the secretory proteins considering similarity search which achieved 23.4% and 26.9% accuracy, respectively. A hybrid-approach that integrated amino acid and dipeptide composition-based modules SVM, and PSI-BLAST, which found increased accuracy 83.2% and sensitivity 60.4% having low 5% false positive predictions). This reflects a substantial increase than achieved using individual modules.

As presented here, versatile applications of AI, MI, and DL in various protein, gene structural, and functional aspects have been evident. Our goal in this article is to go beyond addressing these general features and pinpoint the membrane proteins structures and functions which are addressable using artificial modeling and algorithms. The use of AI, ML and DL techniques is popularly used to understand various features of ion channels. From understanding the amino acid properties and classifications to classifying specific channel subunits representing ion channel families, artificial techniques are utilized [12]. We see that artificial techniques, such as the ML approach, can now capture crucial ion channel complexities related to channel protein expression, correct insertion and folding in membranes, and trafficking to proper locations inside the cell, thus help in further membrane protein engineering and artificial designing [13,14]. AI techniques help us track the early animal evolution by comparative genomics studies of ion channels that specifically help us understand the early evolution of animal nervous systems [15]. ML has recently been used to analyze ion channel genes, especially to extract the feature vectors of various ion channels [16].

It is clear that artificial techniques, models, and algorithms are utilized to program various ion channel features, including classification of channels, channel subunit proteins, or even amino acids and genes, which addresses evolution, modern engineering, and various other related aspects. AI, ML, and DL have a lot of involvement in this new area. Experimental address and their theoretical analysis have produced so much data that we now need these artificial techniques to grasp most about ion channels’ various features in a simplistic manner, using models and algorithms that are made possible using the power of AI, including its subfields ML and DL.

## 2. Bioinformatics Predictions of Ion Channel Structures and Functions

X-ray crystallography, NMR data, etc. on transmembrane proteins are generally used to predict the optimal protein structures. These techniques require the use of extremely expensive necessary ingredients and a tuned laboratory setup. Bioinformatics modeling utilizing appropriate techniques that may promote in silico mechanics and energetics of the protein structure considering the underlying mechanisms are often popularly considered in biophysical studies of proteins. Membrane proteins are generally studied specifically to address their ion channel-forming potency. Bioinformatics techniques play crucial roles when important molecular actions are to be inspected to explain the experimental facts obtained in vitro studies, such as their imaging in the interface of hydrophobic/hydrophilic regions, electrophysiology record of currents across membranes hosting the proteins, etc. Molecular dynamics (MD) simulations often appear as important computational techniques to detect energetics underlying biomolecular interactions. We have been quite successful in biophysical addressing, using MD simulations, of the channel energetics involving channel subunits and membrane lipids for small channels, such as gramicidin A, alamethicin, and chemotherapy drug-induced channels in model membrane systems [17,18,19,20,21,22]. In these publications altogether we could establish a single fundamental fact that the channel stability inside the membrane is due to nothing but molecular mechanisms depending on charge-based screened Coulomb interaction energetics among functional charge groups in the ion channel complex involving channel subunit peptides or drugs and membrane lipids. Our computational in silico assays (numerical computations and MD simulations) simply supported the experimental findings in the distance and time-dependent channel subunit-lipid interaction energetics theoretically. We could calculate the binding energies and evaluate the binding energetics in the channel complex and thus know of the statistical mechanical nature in the channel stability in a biological thermodynamic environment. The readers are invited to read directly from these articles to gain further insights.

Besides various computational assays addressing the general structure and function of channel proteins, bioinformatics templates that draw information from various databank on the channel protein structures, genomics of the proteins, mutations in genes of the ion channel proteins are found to produce crucial information about channel functions in both healthy cells and mutated (disease) conditions.

The aspects addressing the ion channel protein genetics and mutations are presented later in this article using a few example case studies. Here we wish to address the general aspects of ion channel structures and functions using bioinformatics techniques [23] including various computational assays and in silico modeling. Table 1 presents a set of ion channels that are addressed using various in silico computational techniques [24].

A two-decade-old review provided analysis combining MD simulations and various associated calculations with modeling to provide approaches that help understand the structure/function relationships for channels in human cells [25]. Here the modeling techniques were analyzed for potassium channels, the voltage-gated (Kv), and the inward rectifier (Kir) channels. The NMR structures of (the pore-lining) M2 helix were the basis on which the transmembrane region of the pore could be modeled.

What matters to understand the ion channel function is based mostly on two things: (i) ion channel pore region geometry, and (ii) energetics that controls the pore opening/closing phenomena. Direct and indirect experimental techniques usually can address them phenomenologically but underlying mechanisms largely rely on modeling of the channel using bioinformatics techniques [24].

Taking the potassium channel as an example case, Heil and colleagues introduced an interesting bioinformatics method, the so-called ‘Property Signature Method’ (PSM), to address this issue of identification of the channel sequences [12]. This technique relies on physicochemical amino acid properties, instead of amino acid building blocks. A pore region signature (including the selectivity filter) was created, representing the most common physicochemical properties of the known potassium channel, thus enabling the genome-wide screening for the sequences having similar features, despite having low degree of the amino acid similarity within any specific family of the protein.

**Figure 1:**
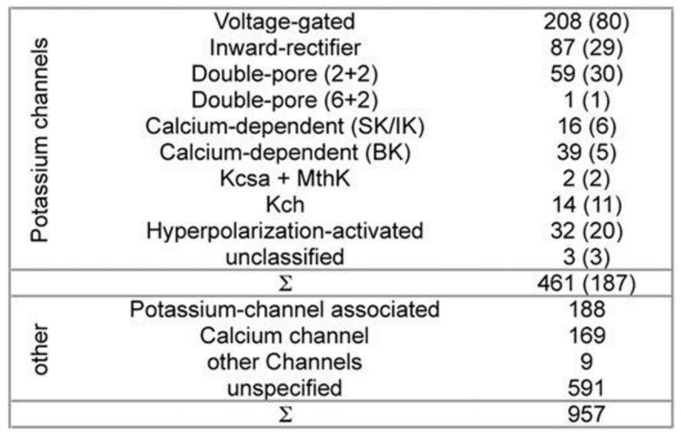
Channel families-the composition of dataset. All of the sequences have been extracted from the Swiss-Prot [26]. Potassium channels represent both the different families and the topologies of the known channels. Non-potassium channels here have been used as the false positives. All of the sequences having >80% sequence-similarity have been removed. The remaining channel numbers are in brackets. The (2 + 2) channels’ double-pore consists of 2 α-subunits having 4 transmembrane domains each. The α-subunits of the (6 + 2) channels with double-pore possess 8 transmembrane domains. Ambiguity exists in 3 unclassified potassium-channels classification.

While developing PSM the dataset used 461 potassium channel α-subunits that represent different family types, see Figure 1 [12]. A pairwise similarity of the sequences <80% were considered (187 sequences). The set was considered to contain additional 957 non-α-subunits, so that false positive could be provided. The sequences included ion channels that are closely related. All of the sequences used here have been extracted from the Swiss-Prot [26].

The pore region profile for a potassium channel was created with the use of the dataset. The profile wasn’t used for describing the conserved positions of the amino acids in the region. But it described all of the variations in various families of the potassium channels. The profile then was translated into creating a descriptor, which describes various sequence region properties. Each profile position located amino acids got analyzed, and the properties with conserved absence or presence were used in order to describe the mentioned position. Here the Hits were ranked following the properties that were found in the property descriptor and in the target sequence. The algorithm of screening was created in the C++ language of programming.

**Figure 2:**
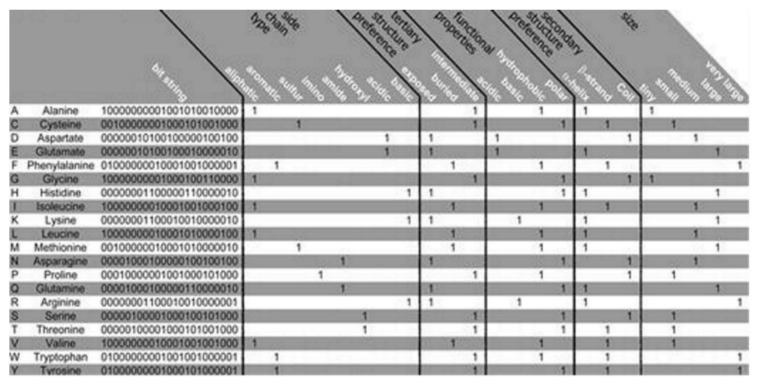
Amino acid properties. The Bit string that represents the amino acids with 23 properties has been presented. The relative occurance frequency got converted into corresponding binary values with the aid of the majority vote. Regarding ‘size’ all of the amino acids got categorized considering the molecular weights: tiny, small, medium, large, and very large for ≤71 Da, ≤103 Da, ≤115 Da, ≤137 Da, and >137 Da, respectively.

The PSM is found to use the representation of the amino acid via consideration of a binary signature that was derived using varieties of physicochemical properties. Altogether, 23 properties were used, combined into 5 groups as follows: the side chain type, the functional properties, the secondary and the tertiary structure (preference), and size, see Figure 2 [12]. Each amino acid is represented by a created binary string, where a bit has been set to 1 for a corresponding property found to apply to the considered amino acid. Five bits have been set, one for every group of the property. Zero is assigned for all of the bits that are remaining. Thus, 20-bit strings (unique) have been found, 1 for every amino acid, which was used in this algorithm. Two steps are considered in the method as follows:

(i) an aligned pore domain profile was created including all of the amino acids that were present (in >3% of the investigated total 461 potassium channels),
(ii) the profile was translated into a representing string consisting of the sequences’ physicochemical properties.

Large-scale potassium channel sequence analysis confirms the requirement of identifying the potassium channel α-subunit proteins [27]. As the family of the potassium channel is found to be highly diverse and also closely related to many other ion channels, the use of the amino acids in order to classify the potassium channels in PSM has been found imprecise. PSM is found superior over Markov models and the BLASTp, see refs. [27,28,29]. Moreover, the PRINTS Database provided potassium channel motifs are used [30]. These approaches are found to utilize multiple methods to overcome a method’s limitations of recognizing the potassium channel family’s subset sequences. These issues are indeed resolved in PSM. Because it can detect properties representing all of the subsets of the family of the potassium channels. Moreover, PSM is able to analyze amino acids’ physicochemically relevant properties and enables pretty sensitive extraction of the information that is coded in the sequences of the amino acids. For details, readers may consult the original article [12].

The *Saccharomyces cerevisiae* genome was well screened applying PSM [12]. Two hits were found, the domains in the pore in the two-pore potassium channel, TOK1, which is the only one known as the S.cerevisiae potassium channel. Despite having a strong relationship including high homology among the potassium transporters, TRK1 and TRK2, to the potassium selective domains of the pore of TOK1, the mentioned two are classified as nothing but the non-potassium channels.

Heil and colleaques also performed another test with Caenorhabditis elegans having a complete genome sequence [31]. Its genome regarding the sequences of the potassium channels is well understood; almost 40 double-pore domains have been annotated. PSM helped recover all. Additionally, a new (potential) pore domain was identified.

**Figure 3:**
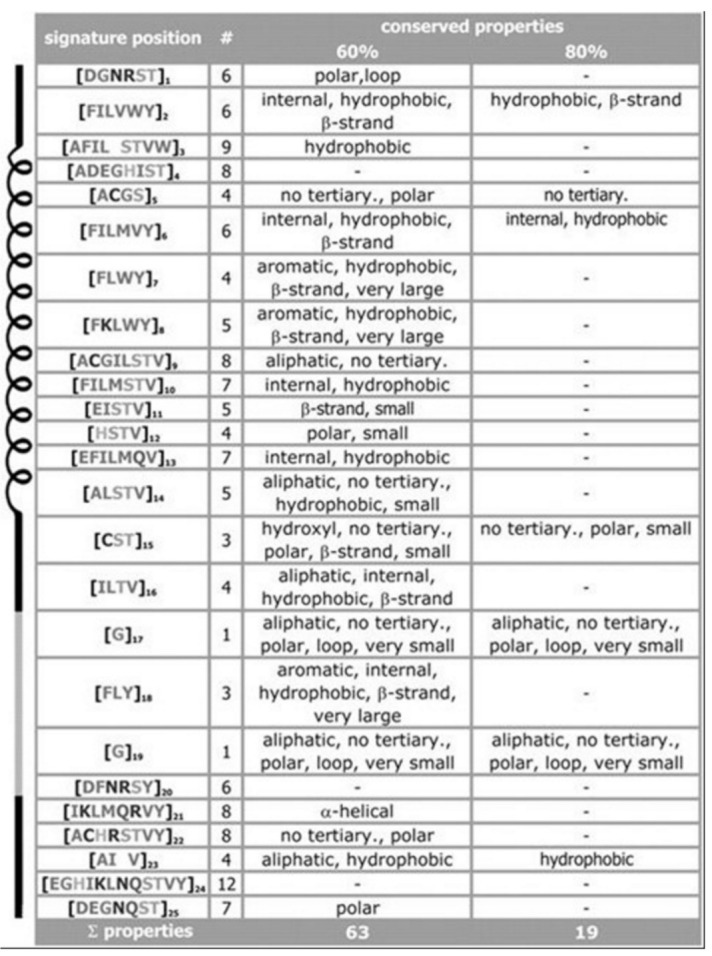
Property conservation at the 60 and 80% level of significance, respectively. Despite having low amount of amino acid conservations we find properties conserved in almost 80% sequences. From pores of the potassium channels, as expected, the hydrophobic residues are found to dominate in pore regions, a few polar residues decrease energetic barriers for K+ ions. Details in ref. [12].

For the signature of the potassium channel, a summary of the conserved properties (at 60% with 80% threshold of conservation) is presented, see Figure 3. Despite considerable sequence set divergency, as many as 63 properties are found conserved with as high as 60% level of significance, and 19 properties are found conserved with as high as 80% level of significance. Unusual properties (not shown) coded in signature; almost 350 properties with 60% level of significance and 330 properties with 80% level of significance. The method specificity draws significant contributions from these mentioned properties.

PSM is considered superior to other conventional methods while searching for the sequences having a pretty low level of conservation. PSM has an important advantage. For every amino acid position, the signature describes the frequent properties (selected and uncommon ones) in the α-subunit portion of the potassium channel. The use of the position-bound signature properties has additional advantages, interpretation of the results appears pretty simple. Next to the missing and unusual number properties, this method is found to return, for every sequence, the display of a vector whose sequence positions are found to contain the untypical and missing residues, respectively, thus facilitating the fast sequence analysis.

## 3. Ion channel Genomes Track the Early Animal Evolution

A comparative study of genomics provides novel windows into the (confusing) past that may be applied for the understanding of the early nervous systems evolution of the animal kingdom [15]. There is a controversy on nervous systems whether they got evolved just once, or independently being distinctive in various animal lineages. Liebeskind and colleagues explored the historical aspects of the gene families of the ion channels, central to the function of the nervous system. They tracked the timeline when the families of the genes expanded in the evolution of the animal and discovered the gene families to be radiated on multiple occasions, occasionally, they underwent various periods of contraction. Multiple gene family origins may be considered to signify considerably the large-scale evolution convergence for the complexity of the nervous system.

The ancestral gene content reconstruction helped was used in tracking the gene family’s expansion timing. Here the majority of the ion-channel protein families that may drive nervous system functions are used. Animals having nervous systems are found broadly to have identical complements of the types of ion channels. But it was also found that these complements could have been evolved independently. Ion channel gene family evolution was found to experience a large amount of loss events, among those two were found to immediately be followed by a few rounds of duplications. Ctenophores, cnidarians, and bilaterians have been found to undergo independent bouts of the gene expansion in the involved channel families connected to the synaptic transmission and the shaping of the action potential, suggesting the genomic signature of the expanding complexity in the nervous system. Ancestral nodes, where the nervous systems probably originated, were found to experience not-so-large expansions. This suggests for the origin of nerves not to experience any immediate complexity bursts, instead, the complexity of evolution perhaps experienced a rather slow fuse in the stem animals, which got followed by gene gains and losses independently.

A custom bioinformatics pipeline [15] was used for collecting and annotating proteins that are predicted in a group of 16 families of ion channels, see Table 2 where 41 sample opisthokonts (this group includes animal, fungi, and related protest members), and an apusozoan outgroup are presented. The channels’ families are found to be playing diversified roles in the nervous systems. Some families (e.g., the families of the voltage-gated ion channels) are found to solely be associated with the function of the nervous systems in the animals, while others (e.g., P2X receptors) are found to play relatively diverse types of roles. Only a handful of isoforms are expressed in the nervous systems. The dataset then got used in order to infer the ancestral contents of the genome and understand the timing of the happening of the gene duplications with the help of EvolMap [32].

**Figure 4:**
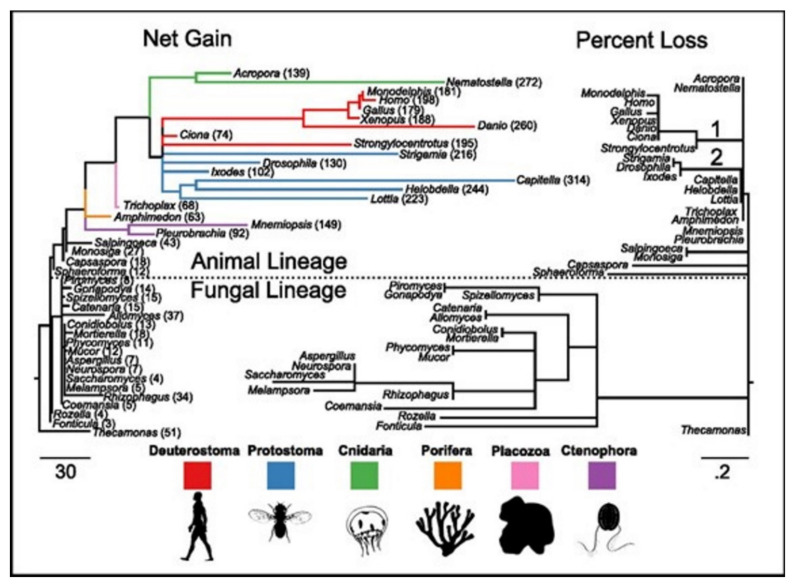
The families of the ion-channels in opisthokont evolution. Both trees contain similar topologies. The lengths of the branch of the left tree are actually net gain, gains-losses. The lengths of the branch of the right tree are the percent loss, losses-gains as a% of the parent copy number. Total ion channel numbers in every taxon are presented on the left tree. Two animal branches having large loss events have been labeled-the common deuterostome and ecdysozoan ancestors.

These gene families were found ancient [33,34]. All except for two, acid-sensing channel (ASC) and the Cys-loop receptor (LIC), are found in the most recent common ancestor (MRCA) of the examined taxa [15]. ASC family was the only one found as the metazoan-specific. The families were pulled together and they then plotted the net gains and the percent losses (on the species tree), see Figure 4 [15]. The animal lineage was dominated by the gains but losses led to the fungal lineage.

In phylogenetic gain and loss patterns for all of 16 families of ion channels (Table 2), large expansions of LIC, voltage-gated potassium channel (Kv), and glutamate-gated channel (GIC) families at multiple places were reported, see details in ref. [15]. This independent gene-(family) expansions lead to MRCAs of the bilaterians, the vertebrates, and the cnidarians [34].

**Figure 5:**
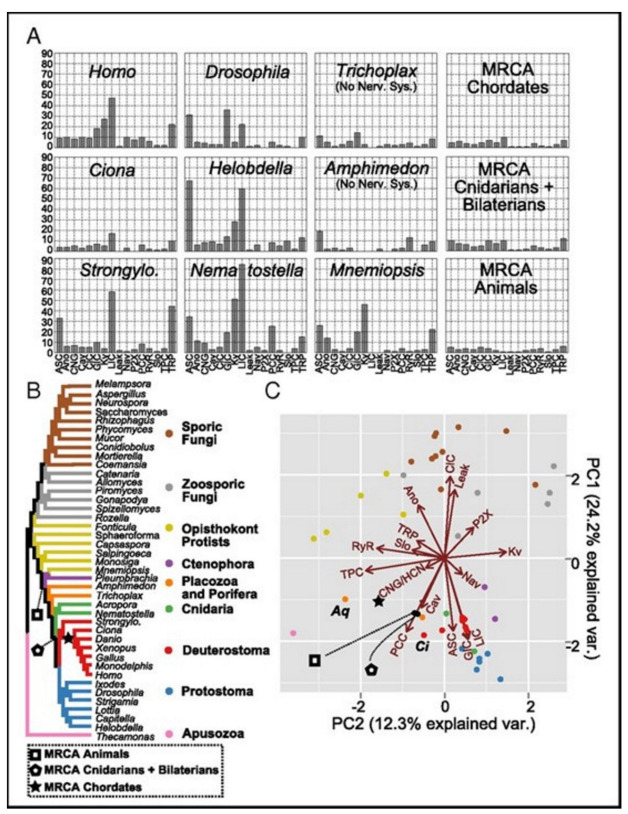
(**A**) The count of the channels of the extant and the ancestral species. (**B**) The species tree shows the relationships between the extant taxa and key ancestral nodes’ locations. (**C**) The PCA of the normalized gene contents of the ion channels for all tips and three ancestral nodes. The proximity in the space of two PCs indicates identical contents of the gene. The ion-channel families loadings have been presented as vectors. The loading vector size and direction indicate its correlation with corresponding two components. The loading arrows are pointing to the regions where the gene family is found in high relative abundance. The labeled species represent Amphimedon (Aq), and Ciona (Ci).

Ecdysozoans and lophotrocozoans were found to have large expansions in LIC, GIC, and Kv channels. A huge expansion in ASC family was also observed, see Figure 5A [15]. These expansions were observed to have happened in terminal lineages that led to every species, see Figure 5. Figure 5A presents the family count of ion channels from species of the major lineage. All taxa with nervous systems, with the notable exception of the tunicate Ciona, were enriched for similar gene families. Two taxa (without the nervous systems), Trichoplax and Amphimedon, were found to have smaller complements of ion channels. MRCAs of the chordates, the cnidarians plus the bilaterians, and the animals each were found to have ion-channel complements resembling the extant animals having no nervous systems more than animals having nervous systems.

## 4. Bioinformatics Prediction of Ion Channel Genes and Channel Classification

Ion channels are indirectly or directly associated with various types of cellular disorders leading to specific diseases. Ion channels are therefore therapeutic and diagnostic targets of many drugs. About 700 drugs are known so far to act upon ion channels [16]. Knowledge of ion channel genes and their mutations certainly is key to understanding diseases and planning for drug discovery. Bioinformatics techniques may be found quite helpful in understanding the roles of ion channels in diseases through analysis of genetics-based classifications [16], as well as genetic mutations [35,36] of ion channels. AI techniques have been found to play important roles in both predicting ion channel genes and understanding genetic mutations and connecting them with classified diseases. We wish to elaborate these features quite in detail here.

### AI Techniques Help Predicting Ion Channel Genes

ML, a subset of AI, was used recently to extract the feature vectors of various ion channels [16]. The SVMProt and the k-skip-n-gram methods were used, which helped obtain 188- and 400-dimensional features, respectively. SVMProt, a web-based support vector machine software, was developed for mainly functional classification of any protein considering its primary sequence [37]. In the case where the structural protein class is inconsiderably correlated with its constituent amino acids, the support vector machine appeared as a computational tool that could predict the structural protein classes [38]. In the k-skip-n-gram method every protein sequence needs to be transferred into a vector. Then the training vectors are used for training the random forest parameters. The testing vectors evaluate the method’s performance.

Various bioinformatics softwares are available to predict the ion channel identifications in membranes. A series of high-throughput computational tools are now available which help predict not only the ion channels but also their types directly using the protein sequences, helping in ion channel targeted drug discovery research. During last decade, many ML algorithm-based computational methods have been developed [39,40], which may be used in drug repositioning. Saha and colleagues used the amino acid and the dipeptide compositions as feature vectors, then classified them with the use of a support vector machine (SVM) so that they could predict the voltage-gated ion channels, and their available subtypes [41]. The identification method for a voltage-gated potassium channel, based on the local sequence information, was also proposed later by another group [42]. The latter is found better than that developed for the identification of the voltage-gated potassium channels, based on the global sequence information [43]. A support vector machine (SVM)-based model was recently constructed which helps predict quickly [44]. A SVM-based model to search the predicted ion channels and subfamilies that uses the sequence similarity search features of the basic local alignment search tools was developed recently [45].

In a recent article, the application of ML Methods in ion channels has been briefed [46]. The review focusses on prediction methods developments for ion channels considering a few issues as follows:

- ion channel proteins datasets,
- predicting ion channels using ML methods,
- obtaining the optimal ion channel prediction features using feature selection technique,
- the prospect of bioinformatics methods prediction of ion channels using appropriate and available tools.

Han and colleagues used SVM and random forest classifiers in order to identify first the ion channels, and further to classify them [16]. The feature selection was made using the maximum-relevance-maximum-distance (MRMD) method that helped improve the accuracy of the prediction. Three steps were followed. Firstly, a protein sequence got detected to check if it might belong to any ion channel. If the positive, then the sequence of the protein got classified as to belong to voltage-gated or ligand-gated ion channels. Finally, if the sequence belonged to the voltage-gated ion channel family, the classification was made regarding them to belong to the potassium (K^+^), the sodium (Na^+^), the calcium (Ca^2+^), or the anion voltage-gated ion channel class.

**Figure 6:**
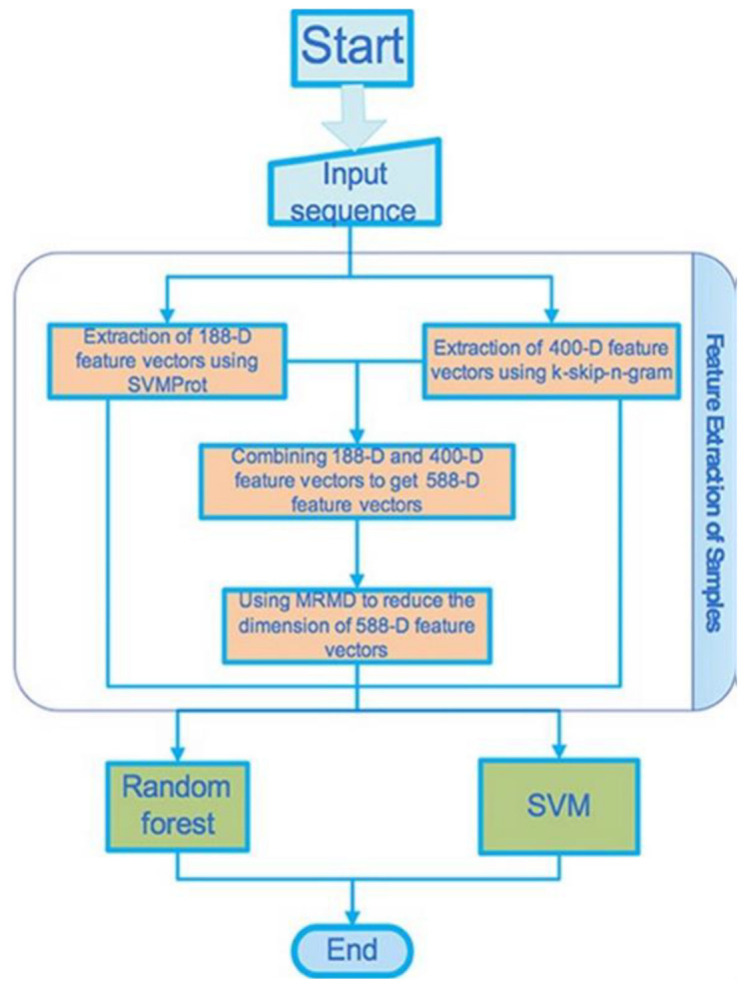
Flowchart representing the proposed processes.

The flowchart shows the stepwise adopted basic processes that Han and colleagues considered for the gene detection and the channel classification, see Figure 6 [16]. We avoid explaining how they introduce the set of data, the method of the feature extraction, the method of the dimension reduction, and the classifier that were used in the study, but the readers may find them in the original article.

The original data used for the prediction model can be found in ref. [43]. The ion channel sequences have been collected from the depository Universal Protein Resource (UniProt) and the depository Ligand-Gated Ion channel databases [46,47]. The total number of the voltage-gated ion channels was 148; 81, 29, 12, 26 of them are potassium channels, calcium channels, sodium channels, the anion channels, respectively. Finally, 150 ligand-gated ion channels were extracted. From the UniProt 300, protein sequences were selected randomly as the non-ion channels, having the consistency of these non-ion channel sequences < 40%. Two ML methods (feature extraction methods), SVMProt 188-D relying on the protein composition and the physicochemical properties, and k-skip-n-gram 400-D were used. These two (feature representation) methods were then combined in order to form a new feature vector that contains multiple (more than one) features. The new feature vector set was then classified using the SVM and the random forest classifiers. MRMD based dimensionality reduction method (see the site http://lab.malab.cn/soft/MRMD/index_en.html, updated by Prof. Quan Zou on 2 November 2016) was then employed for reducing the generated feature vectors’ dimensionality [48]. The MRMD works in selecting the feature having the highest correlation and the least redundancy through calculation of the maximum distance and relevance. Here, they used a random forest classifier for building the model. As this classifier uses multiple trees for training and predicting samples, this one is popularly used in bioinformatics research where applicable, e.g., see ref. [49]. It is found a good performing tool, using especially the random forest algorithm [50], in many practically relevant fields, e.g., the regression and classification of the gene sequences, the action recognition, the face recognition, the anomaly detection in data mining, and the metric learning.

The effects of the prediction of the random forest-based and SVM-based methods on both non-ion and ion channels in various dimensions were compared in this study, see the results in Table 3 [16]. The results for 10-fold cross-validations of 188- and 400-dimensional features and their mixed features have been listed in Table 3. The MRMD method was then applied to reduce 27 dimensions from 588-dimensional features for obtaining 587-dimensional features, with the latter having average classification accuracy lower than that found for the 400-dimensional features. The SVM classifier was reported to be the best to classify the 400-dimensional features. The average overall accuracy (OA) rate, 85.1%. 86.6% of the ion channels, and 83.7% of the non-ion channels, can be identified approximately by the SVM classifier. A total 85.1% accuracy was obtained. Thus feature vectors from 188- and 400- dimensional features yield pretty acceptable prediction results.

The accuracy was evaluated on 188-, 400-dimensional features, and their mixed features, and 88-dimensional features that were obtained following dimensional reduction with the use of MRMD which discriminates between classification results of the voltage-gated and the ligand-gated channels. All these results are summarized in Table 4 for these two classes and in Table 5 for ion specificity in voltage-gated ion channels [16]. 93.9% and 86.0% of the voltage-gated and ligand-gated ion channels, respectively, could correctly be identified with the use of the random forest method. This classifier is a better performer than the SVM classifier especially in a few cases, and also can provide an improved prediction performance model.

## 5. Detection of Ion Channel Genetic Mutations Using AI Techniques

Mutations in genes are generally known to be responsible for diseases. Genetic mutations involving ion channel subunits or proteins may also often be found responsible for various diseases. AI techniques may be applied to establish such evidence in bioinformatics explorations. We shall use a few case studies to address this phenomenon for certain diseases.

### 5.1. Ion Channel Genetic Variants in Epilepsy

The mutations of ion channels are known to raise causes for rare Mendelian disorders affecting the heart, the brain, and various other tissues. Mendelian mutations have been found linked with various single-channel defects that cause the familial episodic and the degenerative excitability disorders of the cardiovascular [51], the nervous [52], the neuroendocrine [53] and the immune surveillance systems [54].

Klassen and colleagues did parallel exome sequencing on 237 genes of channels in the human sample. They compared the variant profiles of the unaffected individuals to the individuals having the most common disorders related to neuronal excitability, sporadic idiopathic epilepsy [35]. A rare missense variant in the known Mendelian (disease) genes was reported, prevalent in both groups with identical complexity. Thus it proves that even the deleterious channel mutations may confer uncertain risks to any individual, depending on other variants they are combined with.

Comparisons were made on the polymorphism (SNP) profiles of the exomic single nucleotide, including the type, the relative burden, and the variants pattern within a large number of genes of the ion channel candidate, set between healthy/unaffected individuals and ones with severe disease of the neurological excitability in order to evaluate the personal genetic liability. Table 6 summarizes SNPs [35].

**Figure 7:**
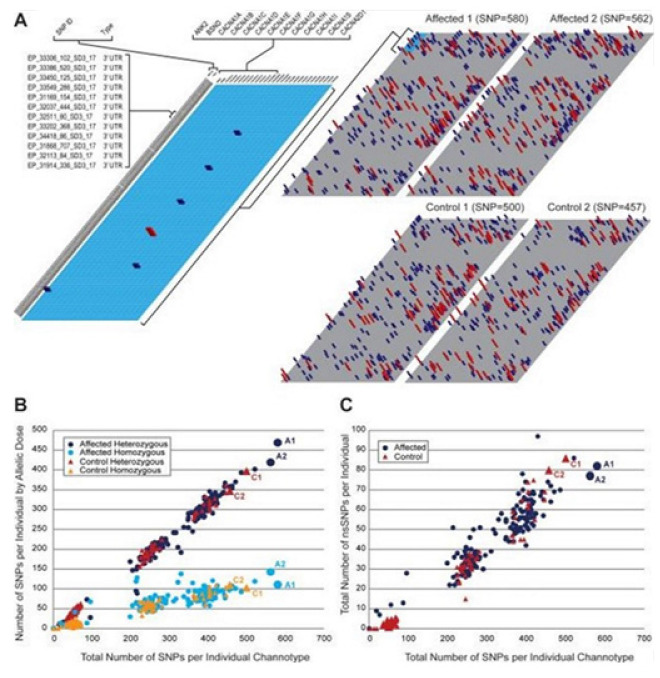
All variants are to render unique channotype. (**A**) The low resolution (in gray color background) 3D representation illustrates the extreme channotypes that are present in the cohort study (2 cases and 2 controls, each having >450 SNPs). Columns are to list the genes of the channel subunits in an alphabetical order (ANK—SCN), and rows are to list the validated individual identifiers of the SNP organized in alphabet order by the type (3′UTR—promoter). The enlargement at the left in the teal is presented here for the clarity and the scale. The dosage of the gene of minor (variant) allele for a SNP is denoted here by the bar (tall red = Homozygous Minor Allele; short blue = Heterozygous Minor Allele). Sparsely populated regions present in all four channotypes reflect low frequency novel SNPs. (**B**) The histogram for all the individuals by the cohort with total SNP number in individual plotted here against the total SNP number in heterozygous or homozygous channotype. The affected and the control cohorts are found to show identical dosages of the allelic with the increasing count of the SNP. (**C**) The histogram of all of the individuals within every cohort show the total SNP number per individual plotted against the nsSNP number contained in the channotype. The nsSNP number in a channotype increases with the total SNP count increase in both of the populations. The individual channotypes profiled in A. (A1,A2,C1,C2) are indicated in histograms.

The study claims SNPs for every targeted gene in both groups; of the validated SNPs, 1355 were unique to either population, and their majority, 1740, was shared. The data have expanded the list of the known channel SNPs in dbSNP. This addition also confirms the rare allelic variation across a lot of genes of the ion channels. The huge variation is found to agree to those that emerged from the whole-genome sequencing of the individuals [55], from >2100 cases screened for the variants in (the clinically important) cardiac channel gene subset [56]. Any individual channotype is unique. In the cohort, no individuals were free of SNPs, and no two channotypes from 291 individuals were found identical, see Figure 7 [35]. An overlapping SNP type variety was found in both groups, which include sSNPs, nsSNPs, and SNPs in the promoter, coding, UTR, and intronic regions. Both populations contain nonsense SNPs.

**Figure 8:**
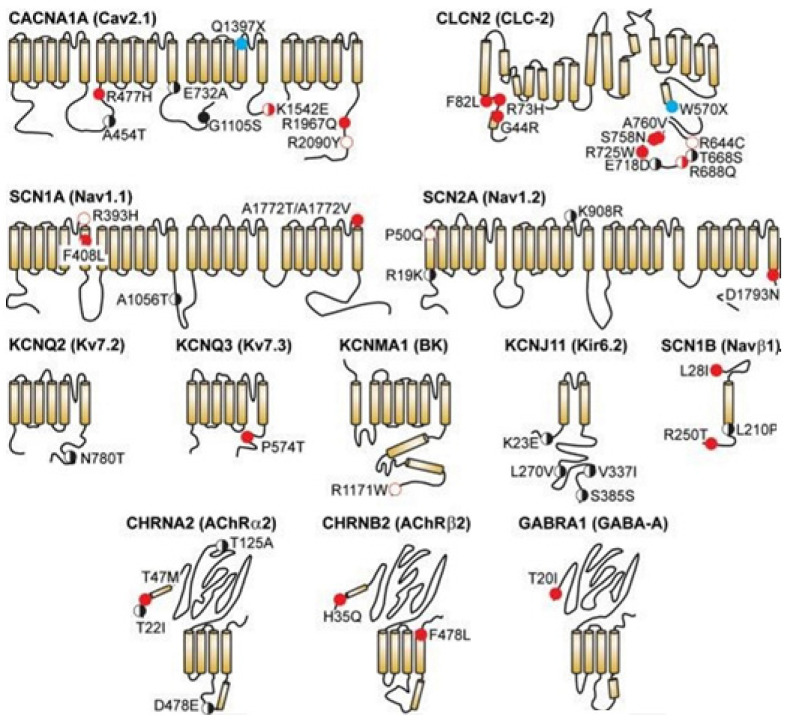
The known genes population of the monogenic human epilepsy (hEP) with the missense and the nonsense variants found in cohort. The products of the protein of 12 ion channel genes known to be causing the monogenic epilepsy have been shown here schematically. The validated missense, and the nonsense SNPs that are discovered through profiling have been represented here by circles that mark the nearest amino acid location, determined by the comparative multiple alignment. The presence of a SNP denoted by the fill pattern (the filled circle = in affected only; open circle = in controls only; half-filled circle = SNP is present in both of the groups). The nsSNPs in dbSNP have been colored in black, novel nsSNPs from the study are colored in red, and the nonsense SNPs have been colored in blue.

300 missense channel variants were detected in 139 (unaffected) individuals. 23 are in human epilepsy (hEP) genes that signal that the allelic penetrance in channelopathy is underappreciated, see Figure 8 [35]. R393H nsSNP in SCN1A gene’s ion-selective pore perhaps causes severe myoclonic epilepsy in infancy [57], detected once, and in only a control population. The in vitro studies however failed to record sodium currents [58] indicating that the protein structural deleterious alterations in the known hEP gene are not sufficient so that the risk of epilepsy can be detected.

The study also found the value of the computational models in assisting in the personal risk predictions [35]. Idiopathic epilepsy (IE), with no cause known yet, had been found as an ideal condition in order to study sporadic genetic channel variation’s impact on cortical function, as seizure disorders affect 1–2% population. Analyses of rare Mendelian forms revealed that the ion channels are the major phenotype determinants, as 17/20 (confirmed) monogenic syndromes are found to arise in individuals that are heterozygous for any SNP in a gene in the channel subunit [59]. Thus, the study observed considerable genetic complexities.

Understanding the genetic mutations in ion channels using bioinformatics techniques is expected to help largely in drug discovery. In epilepsy, almost a third of patients are found refractory to the current anti-epileptic treatments of drugs. With few exceptions, they target the ion channels. The sequence variants, which alter access to the binding sites of the drugs, are obviously the candidates with mechanisms thereof for pharmacoresistant. The variant profiles perhaps personalize the treatments through identification of the ineffective drugs for epilepsy and various other excitability bourn disorders that concern modulations of channels. Thus these profiles are found clinically useful.

### 5.2. Ion Channel Genetic Variants in Alzheimer’s Disease

Alzheimer’s disease (AD) is known as a heterogeneous genetic disorder that is characterized by early hippocampal atrophy and the cerebral deposition of the Aβ peptide. The Tissue Info (for screening genes found to get expressed preferentially in the hippocampus, located in AD linkage regions) was used for discovering a novel gene on 10q24.33, called CALHM1 [60]. CALHM1 encodes a multipass transmembrane glycoprotein that controls cytosolic Ca^2+^ concentrations and Aβ levels. CALHM1 homomultimerizes, shares considerable sequence similarities with the NMDA receptor’s selectivity filter and generates a considerable Ca^2+^ conductance across the plasma membrane. It was determined that the CALHM1 P86L polymorphism (rs2986017) is significantly associated with AD in independent case-control studies of 3404 participants (allele-specific OR = 1.44, *p* = 2 × 10^−10^). The P86L polymorphism was found to increase Aβ levels by interfering with CALHM1-mediated Ca^2+^ permeability. Thus a conclusion was made that CALHM1 perhaps encodes an essential cerebral Ca^2+^ channel component that may control the Aβ levels and the AD susceptibility.

Dreses-Werringloer and colleagues showed that a CALHM1 structural region shares the sequence similarities with NMDAR’s selectivity filter and that the N72 residue is a key determinant in the control of cytosolic Ca^2+^ levels by CALHM1 [60]. Electrophysiological study on CALHM1-expressed in Xenopus oocytes and CHO cells was found to reveal CALHM1 to induce a novel Ca^2+^ selective cation current across the plasma membrane. This suggests that CALHM1 may cause the construction of a novel pore/ion channel, for details see [60]. In a subsequent study, however, the rare CALHM1 genetic variants got reported which may lead to the Ca^2+^ dysregulation and predicted to perhaps contribute to the risk of EOAD through some mechanism that is independent from the classical Aß cascade [61]. All CALHM1 coding regions in three independent series comprising 284 EOAD patients and 326 controls were sequenced. 2 mutations in missense, p.G330D and p.R154H, and a p.A213T in an individual control have been identified. Calcium imaging analyses revealed that while the mutation found in a control (p.A213T) behaved as wild-type CALHM1 (CALHM1-WT), a complete abolishment of the Ca^2+^ influx was associated with the mutations found in EOAD patients (p.G330D and p.R154H). The CALHM1 P86L polymorphism was found in another study associated with elevated cerebrospinal fluid (CSF) Aβ in normal individuals at risk for AD, which indeed support that CALHM1 controls Aβ metabolism in vitro in cell lines [60] and in vivo in human CSF [62]. Here despite having crucial molecular level understanding in mentioned various findings, we indeed wish to elaborate on understanding the genetic mutations in ion channels concerning AD utilizing Bioinformatics techniques [60].

**Figure 9:**
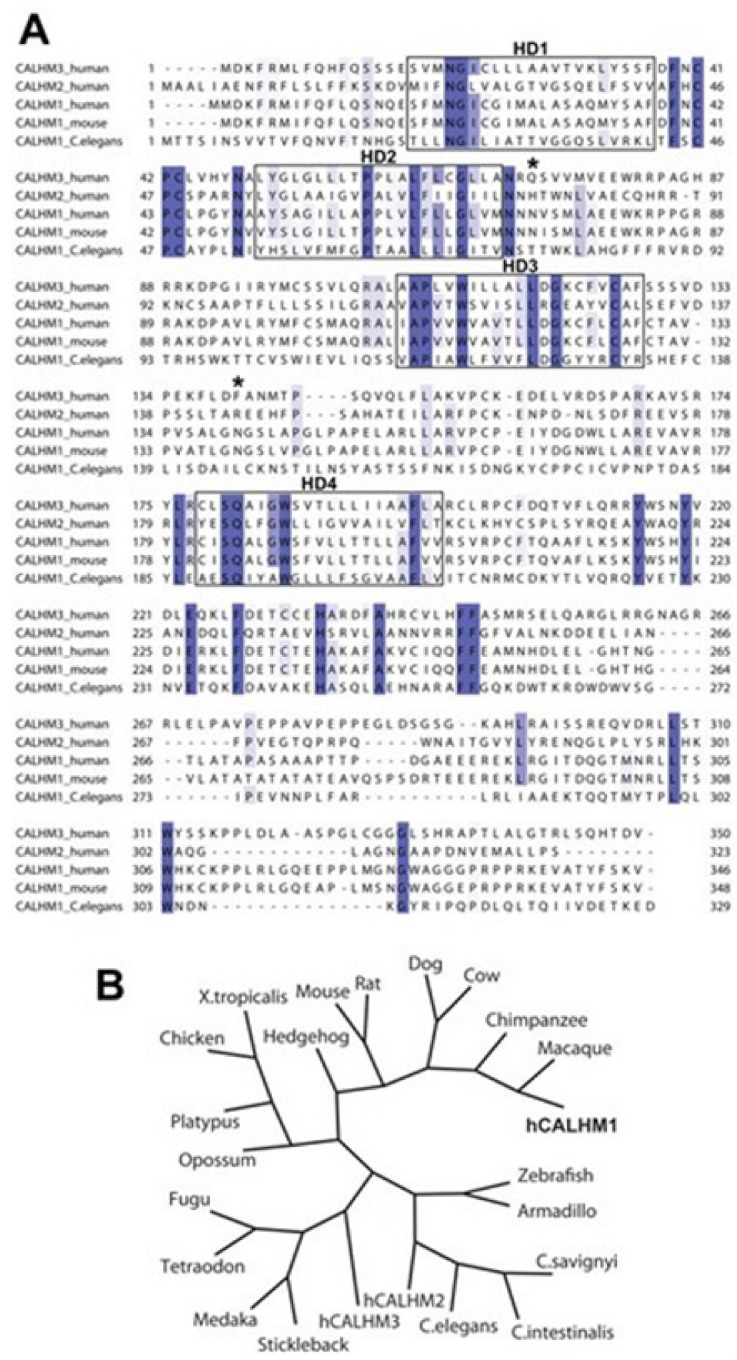
The alignment and the phylogeny of the CALHM1. (**A**) the alignment of the sequence of the human CALHM3, CALHM2, and CALHM1, and of the murine and the C. elegans CALHM1. The conserved sequences have been highlighted with blue and the sequence conservation has been mapped with a gradient of color, the darkest color is used to represent the sequences having absolute level of identity and the lighter colors to represent the sequences having weaker level conservation. The boxes are to denote the hydrophobic domains 1–4 (HD1–4). Stars, the predicted sites of N-glycosylation on the human CALHM1. (**B**) the phylogenetic tree that include the human CALHM1, denoted as ‘hCALHM1′.

In ref. [60], the human genome with TissueInfo (a pipeline of bioinformatics that helps calculate the profile of tissue expression) was studied to annotate the human transcripts having the expression levels of the tissue derived from the database-expressed sequence tag database (dbEST) [63]. TissueInfo screen was found to identify 30 transcripts (from 33,249 human transcripts), corresponding to the investigated 12 genes, having hippocampus expression, see Table 7 [60]. These transcripts were found to match either of the two hippocampus sequenced ESTs. One unknown gene function, which was previously annotated as FAM26C, was found to match two ESTs of the hippocampus and found to be mapped to AD locus on the 10q24.33. This gene CALHM1 (calcium homeostasis modulator 1) is known to encode the open reading frame (ORF) of the amino acids (346 altogether) and is mainly predicted to have a structure containing 4 hydrophobic domains (HDs; TMHMM prediction), and 2 N-glycosylation motifs (NetNGlyc 1.0 prediction) (Figure 9A). the search of the sequence database identified 5 (human) homologs of CALHM1, identified collectively as the family of FAM26 gene. 2 human homologs CALHM1 with the broader profiles of the tissue expression, are clustered to next of CALHM1 in the 10q24.33 and designated CALHM2 having 26% of the identity of the protein sequence, annotated previously as the FAM26B, and CALHM3 with the identity score 39%, FAM26A (Figure 9A). CALHM1 is conserved across at least 20 species, including mouse and C. elegans (Figure 9A,B).

CALHM1 maps to the chromosomal region that is associated with the LOAD susceptibility, tested to see if CALHM1 SNPs could be found associated with the disease development risks.

2 non-synonymous SNPs have been found in databases, rs2986017 (+394 C/T; P86L) and rs17853566 (+927 C/A; H264N). Dreses-Werringloer and colleagues did sequence the entire ORF of CALHM1 with the use of the genomic DNA considered in 69 individuals, that include 46 (autopsy-confirmed) AD disease cases and 23 (age-matched) controls [60]. rs17853566 SNP has not been observed here, rs2986017 SNP presence has been confirmed, having an over-representation potential of T allele in the AD subjects (with AD account for 36% and controls 22%), presented in Table 8, details in ref. [60]. rs2986017′s impact on the AD developing in 4 other (independent) control-case populations (2043 Ads, 1361 controls combined, presented in Table 8) was then tested. The distribution of the T allele was increased for ADs over that for controls for all studies, having the odds ratios (ORs) to range between 1.29–1.99 (here OR = 1.44 and *p* = 2 × 10^−10^ for combined population). The association has been found highly homogeneous among all tested case-control studies, tested for the heterogeneity: *p* = 0.59 and I^2^ = 0%. T allele frequency in (autopsy-confirmed) ADs was found similar to the values observed for the probable populations of the AD case (Table 8). For combined population, CT or TT genotypes have both been found associated with the enhanced risk of AD development (ORCT vs. CC ranges between 1.18–1.64 with OR = 1.37, *p* = 3 × 10^−5^ for the combined case of population, and ORTT vs. CC between 1.44–4.02 with OR = 2.03, *p* = 2 × 10^−7^ in combined population). APOE status wasn’t considered in all observations (Table 8, and *p* with interaction = 0.26).

In the report, a compelling piece of evidence is revealed that rs2986017 SNP in CALHM1, which results in the substitution of the P86L, is actually associated with the increased risk for LOAD and significant Ca^2+^ homeostasis dysregulation and APP metabolism. The P86L polymorphism was found to impair the permeability of the Ca^2+^ in the plasma membrane, reduces the cytosolic Ca^2+^ level, affects the production of sAPPα, and cause concomitant derepressing of CALHM1′s effects on the Aβ accumulation. Indeed, these results help progress in understanding AD involving ion channel malfunctions due to specific genetic mutations, thanks partially to bioinformatics techniques, the establishment of various databases, and the development of advanced algorithms.

## 6. Deep Learning Models Explain Ion Channel Features

Earlier we have addressed how ML can help understand crucial ion channel aspects. Here we wish to familiarize the role of another popular technique Deep Learning (DL) in understanding ion channels. Application of ML algorithms (e.g., in ion channel understanding) almost always requires structural (e.g., ion channel protein) data, while DL networks rely on layers of artificial neural networks. Both ML and DL are actually forms of AI, although DL is considered a specific kind of ML. Both of these AI techniques start with the training, and test the data and a model, then proceed with the process of optimization to ultimately search for the weights which make this model fit best to the data. In this section, we wish to see how DL may assist us in a developed understanding of ion channels. We must keep in mind that ion channel understanding using this new AI technique is just celebrating its beginning. So readers, though will get an introduction, may not get any fully conclusive scenario related to crucial ion channel structural and functional aspects.

### 6.1. Deep Learning Model Idealizes Single Molecular Activity of Ion Channels

A DL model considering the convolutional neural networks and the long short-term memory architecture has just been found. It automatically idealizes the complex activity of the single-molecule with enhanced accuracy and that the process is pretty fast, for details see ref. [64]. The critical first step in understanding the electrophysiology technique recorded ion channel current traces lies in event detection, which is the so-called “idealization”. Here the (noisy) raw data have been are turned to the discrete protein movement trends [65,66]. But till today enormous practical limitations are faced in the idealization of the patch-clamp data. The highly acceptable, or quality idealization is found typically quite laborious, becomes infeasible, subjective with the complex biological data that contain various distinct native (single-ion channel) proteins’ simultaneous gatings. In the DL model of Celik and colleagues, there are no parameters to set; baseline, channel amplitude or numbers of channels for example. This DL model may therefore be useful in getting an unsupervised and automatic detection of the transition events of the single molecules.

Both the fluorescence resonance energy transfer (FRET) and the patch-clamp electrophysiology on single-molecule research are known to provide real-time data on the molecular protein state with high resolution. But the data analysis is usually very time-consuming, laborious, and requires expert-level supervision. Celik and colleagues have demonstrated that an automated event detection in patch-clamp data is possible using the deep neural network, and Deep-Channel, combining recurrent and convolutional layers. This relatively easier method is found to work with enhanced accuracy over a considerable amount of input datasets.

A hybrid recurrent convolutional neural network (RCNN) model [64,67] is introduced to idealize the records of ion channels, up to 5 channel events that occur simultaneously. For training and validating the models, another analog-synthetic ion channel record system generator was developed and it has been found that the our Deep-Channel model, involving long short-term memory (LSTM) and convolutional neural network (CNN) layers, idealizes rapidly with high accuracy, or detects the experimentally recorded single molecular events without the necessity of the human supervisions.

**Figure 10:**
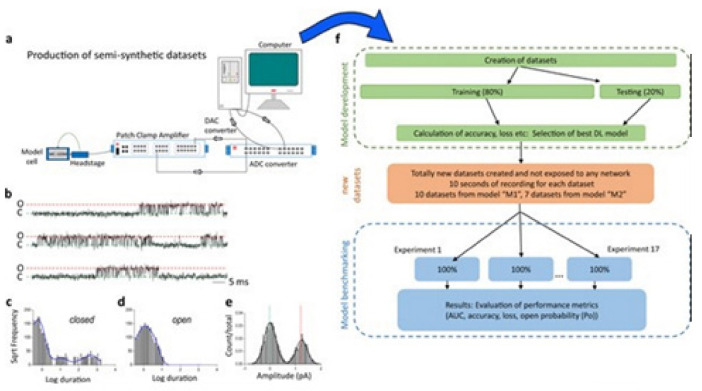
The workflow-diagram: the artificial analogue datasets generation. (**a**) the training, the validation and the benchmarking, the data got generated first as the fiducial records with having authenticated kinetic-models using MATLAB (Figure 11); the data have then been played out via a CED digital-to-analogue converter to the amplifier of a patch clamp sending the signal into a (model) cell, and then recorded the signal back (simultaneously) to a hard disk having the CED Signal software through a CED analogue-to-digital converter. The noise degree got altered by moving the headstage of the patch-clamp closer to, or a bit further from PC. Raw patch-clamp data produced in these described methods are found indistinguishable from the genuine patch-clamp data. For illustrating the point, this shows a standard work-up analysis for an experiment having (**b**) raw data, followed by it’s analyses with QuB: kinetic analyses of (**c**) channel-open and (**d**) closed dwell times. They finally show (**e**) all the points amplitude histogram (**f**).

**Figure 11:**
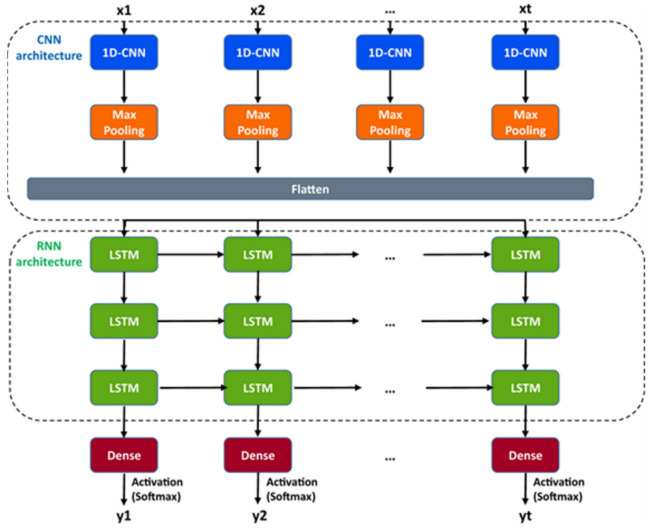
The data of the input time series were fed to the layer of the 1D Convolution (1D-CNN) including 1D convolution, and max pooling layers. Then the data was flattened to next network layer shape, an LSTM. 3 LSTM layers got stacked, each containing 256 LSTM units. The dropout layers got appended with all of the LSTM layers, having the value 0.2 to reduce overfitting. This returned features from the stacked LSTM layers. For additional details of the flowchart readers may go through the ref. article.

**Figure 12:**
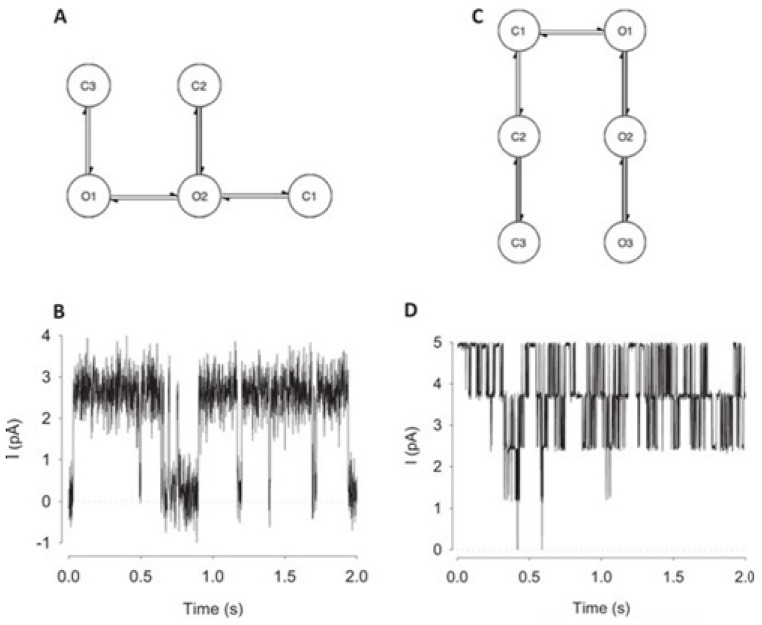
“Patch-clamp” data were produced from two different stochastic models. (**A**,**C**) The Markovian models simulating the ion channel data. Ion channels move between closed (0 conductance) and open (unitary-conductance, g) states. In many cases, there are many open and closed states (“O1”–“O3”, or “C1”–“C3”, respectively). The central dogma of ion channel research is that the g will be the same for O1, O2, or O3. Although substates have been identified in some situations, these are beyond the scope of our current work. (**A**) Model M1; the stochastic model from ref. [68] and its output. (**B**) It has a low open probability, so the data is a representation of the 0 or a channel openning state. (**C**) Model M2; the stochastic model, and an output data (**D**) since the open probability is high, the signal is found to be largely composed of 3 or more ion channels that are simultaneously open.

**Figure 13:**
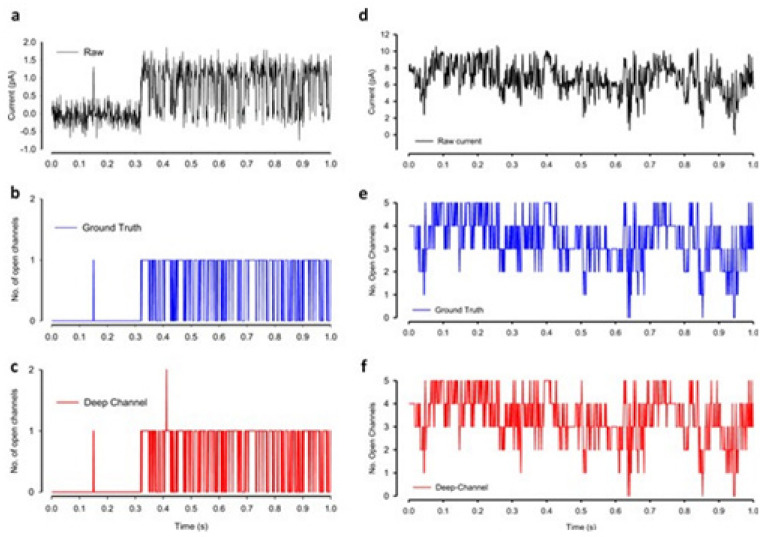
The qualitative performance of the Deep-Channel with (previously) unseen data. (**a**–**c**) represent examples of Deep-Channel classification performance with ion channels having low activity (data from model M1, Figure 13a,b): (**a**) raw semi-simulated data of the channel event (in black). (**b**) ground truth idealisation/annotation labels (in blue) from the raw data in (**a**). (**c**) Deep-Channel predictions (in red) for the raw data (**a**). (**d**–**f**) representative examples of Deep-Channel classification performance with five ion channel openings simultaneously (datafrom model M2, Figure 13c,d). (**d**) semi-simulated raw channel events data (black). (**e**) ground truth idealisation/annotation labels (in blue) using raw data in (**d**). (**f**) the Deep-Channel label predictions (in red) for raw data (**d**).

Figure 10 illustrates the data generation workflow and Figure 11 illustrates the Deep-Channel architecture [64]. Whilst the LSTM models were found to give a good level of performance, its combination with the time-distributed CNN was found to give higher or increased performance. This RCNN was so-called Deep-Channel by Celik and colleagues. Following the training and the model development, see methods in ref. [64], 17 generated new datasets were used, unseen previously by the Deep-Channel, thus uninvolved in training processes. Authentic channel data, see Figure 10b, have been generated. Two kinetic schemes, the so-called first (M1) having low ion channel opening probability, and the so-called second (M2) having a high channel opening probability were applied, and thus an average of the approximately 3 channels open at a time was obtained (Figure 12b). Examples of the data, with the ground truth and the Deep-Channel idealization, have been shown in Figure 13 [64]. All of the Deep-Channel results described here have been achieved without requiring any human intervention beyond providing the script with the correct name of the file or the path.

Illustrates their overall model designing and the testing workflow. The provided Supplementary Information [64] includes training metrics from the initial validation point and the main text shows the performance metrics that were acquired from the 17 experiments having novel datasets. In training datasets, there were typically contained millions of sample points, and the 17 benchmarking experiments were sequences of the 100,000 samples each.

For channels having a relatively low channel-opening probability (see stochastic gating model M1, Figure 12a), the data idealization process is found to get close to a binary-detection problem (see Figure 13a), having the channel events’ type closed/open, labels “0”/“1”, respectively. Here, the so-called receiver operating characteristic (ROC) curve, applied for the classification of the channel events for open and closed state detections exceeds a high level of 96%. In low channel open probability case of experiments, the Deep-Channel was found to return a macro-F1 of 97.1 ± 0.02%, but the segmented-k means (SKM) method in the software package QuB was found to result in a macro-F1 of 95.5 ± 0.025%, and 50% threshold method in QuB gave a macro-F1 of 84.7 ± 0.05%, n = 10.

For datasets including the highly active ion channels (from the model M2), we get it to becoming a multi-class problem of comparison, hence the Deep-Channel was found to outperform both 50% threshold-crossing and the SKM methods in QuB quite considerably. The Deep-Channel macro-F1 was 0.87 ± 0.07, however SKM macro-F1 in QuB, without the manual-baseline corrections, was found to drop sharply to a value 0.57 ± 0.15, and the 50% threshold-crossing macro-F1 was found to fall to a value of 0.47 ± 0.37 (the student’s paired t-test between methods, *p* = 0.0052).

### 6.2. Deep Learning to Classify the Ion Transporters and the Channels from the Membrane Proteins

Recently, an article was published proposing a DL method for automatic classification of the ion transporters or pumps and the ion channels from the membrane proteins [69]. This technique is proposed through training the deep neural networks and by using the position-specific scoring matrix profile used as the input.

From structural and behavioral perspectives ion channels are found to differ significantly from ion transporters, see ref. [69] (reproduction of the figure is not granted). The DL method of Taju and Ou is dedicated to distinguishably classifying these two structural events. Three-stage approaches have been adopted, where 5 techniques of data normalization have been used; the next 3 imbalanced data techniques have been applied for the minority classes, then 6 classifiers have been compared to the method proposed here, for details see original article [69]. We shall present here a brief of the results and interpretations.

The goal here is to find a method that will be able to automatically classify the ion transporters and the ion channels from a set of membrane proteins through training the deep neural networks (DNNs) that uses a convolutional neural network (CNN) as its selected algorithm capturing the hidden pattern of information in the set of data. The hidden feature that is extracted in the position-specific scoring matrix (PSSM) from the data set of proteins is thus expected to be the best feature producing the relevant evolution information related to the sequences of the proteins. More importantly, the feature obtained here should be applicable to versatile problems in the fields of bioinformatics and ML, with considerable and promising outcomes or results, when compared to other available feature extraction methods. Firstly, all protein data representation in the format of FASTA (stands for fast-all) is changed into another PSSM profiles’ format. Secondly, DL, demonstrated by the use of such representation will be able for accurately classifying some proteins separated from the data for training. Lastly, for validating this approach, 5 cross-validations are used and test the proposed method’s modeling.

The guidelines of the 5-step rule [70] are followed for making following 5 steps clearly:

- the method of constructing or selecting a valid benchmark data set for training and testing the predictor;
- the method of formulating biological-sequence samples with the help of any effective mathematical expression which can accurately reflect their intrinsic correlation to the to-be-predicted target;
- the way on introducing or developing a powerful algorithm or engine for operating the prediction;
- the way on properly performing the tests for cross-validations for objective evaluation of the anticipated predictor accuracy;
- the way on establishing a friendly to the users’ web server for predictor which will be accessible to the mass public.

For details on all these five steps, readers may see ref. [69]. In ref. [69] (reproduction of the figure is not granted), a schematic representation of the membrane protein classification prediction steps has been provided. The dataset used is collected from the database of the Universal Protein Knowledgebase (UniProtKB) (accessed on 14 April 2018) (UniProt, 2016) (see Table 9).

We avoid elaborating on the detailed techniques. To represent the input data, a PSSM-based feature extractor was applied here and a 20 × 20 matrix was produced. Initially, the Position-Specific Iterative Basic Local Alignment Search Tool (PSI-BLAST) [28] against (ftp://ftp.ncbi.nih.gov/blast/db/FASTA/) (31 August 1997) was used for generating the PSSM profiles. The PSI-BLAST is a method for searching the protein sequence profile and the PSSM is the matrix generated utilizing a protein query that can perform PSI-BLAST search for finding its similarity from biological databases, and thus creates the (position-specific) matrix. For every query of protein, PSSM can produce N × 20 matrix having a component of the profile, where N denotes the protein sequence length and columns represent the scores for the substitution of the amino acids in the protein. For details, see ref. [69].

The 20 amino acids composition analysis, the n-gram analysis, the sequence motif visualization with the use of the word cloud technique have been shown. The 20 amino acid residues variance has been computed considering the 3 protein data set classes. The experiments compared the DL model performance against 5 different techniques of data normalization and 3 techniques for oversampling. The model was evaluated using the k-fold cross-validation. The best performance of the model was then compared with a few classifiers, such as the Perceptron Gaussian Naïve Bayes, the Random Forest, the Nearest Neighbors, the SVM, and the Nearest Centroid classifiers with the use of independent test data for examining different algorithms’ effects. The analysis of the sequence got performed on the platform of the training data for finding a little information on the amino acids and base pair of the residue patterns at the important motifs in the sets of the data. In ref. [69] (reproduction of the figure is not granted), we see the amino acids having letter Ala (A), Gly (G), Leu (L), Ser (S), and Val (V) have been dominant and also important, as we see in amino acid composition figure or the amino acids occurrence frequency in all of the proteins. The 20 amino acid residues variance across ion channels, ion transporters, membrane proteins have been computed. The variance analysis got used for measuring the data spreading distance away from the overall average value. Amino acids Leu (L), Ser (S), Ala (A), Val (V), Gly (G), Glu (E), Ile (I), Arg (R), and Thr (T) with high frequencies in analysis top frequent motifs showed a variance value below 0.005, and Cys (C), Lys (K), and Trp (W) showed a variance value above 0.005 of 0.013, 0.012, and 0.032, respectively.

The datasets were then classified to distinguish three classes, namely ion channels (class A), ion transporters (class B), and other proteins (class C) using the following techniques, for details (not presented here) see ref. [69]:

- Comparative results were extracted using different techniques for feature normalization
- Comparative results on different techniques for the imbalanced data set

To evaluate the predictor model performance, 5-fold cross validations have been applied in training data sets. Table 10 reports the results of the fivefold cross-validation technique that was applied in the training data, which is a challenging step to find the best model prediction of independent test sets. The performance is seen to reach the highest Sen (89.20%), Spec (84.89%), Overall Acc (87.05%), and MCC (0.75) for class A. Class B achieves Sen (86.76%), Spec (88.23%), Overall Acc (87.49%), and MCC (0.75), and performance of Sen (92.50%), Spec (96.19%), Overall Acc (94.35%), and MCC (0.89) are seen for class C.

The application of all these Deep-Channel algorithms and models has been found possible, though with limitations, for the case of biological data on ion channels. We have presented here basically two example studies where DL algorithms have been utilized to demonstrate various ion channel features. The effectiveness of Deep-Channel to detect events in the single-molecule datasets has been mainly demonstrated. The method is exclusively applicable not only for patch-clamp experimental data, but it has potential for the deep learning convolution or LSTM networks for tackling other related biological data analysis problems. The ion channel, ion pump, and other membrane protein classification using DL algorithms and modeling has been found quite impressive and time and resource saving initiative. Over the next decade, we may see an exponential increase in use of AI, ML, and DL in understanding natural status and mutated conditions of ion channels of biological cells.

## 7. ML in Ion Channel Engineering

AI techniques are found helpful in ion channel engineering. Recently, ML is reported to help in designing the membrane hosted channelrhodopsins (ChRs) for the eukaryotic expression and the plasma membrane localization efficiently [14]. Here a predictive ML approach has been used that can capture the complexity and facilitates successfully the MP design and engineering. The application of ML on the training sequences that are made by the structure-guided SCHEMA recombination enables to predict accurately the rare sequences in a library of pretty diverse members of channelrhodopsins (ChRs), expressed and localized to the mammalian cells’ plasma membranes.

In protein engineering, membrane protein (MP) sequence changes, influencing expression and membrane localization, are highly context-dependent. That means what changes are found to eliminate localization in one sequence context may have almost no effect in another. Subtle amino acid changes may have dramatic effects [14,71]. It is conclusive that the MP sequence determinants of expression and membrane localization are not necessarily captured by ordinary rules as applied in studies [72] providing information on signal peptide sequence having positive charge at the membrane–cytoplasm interface “positive-inside” rule [73], and an enhanced hydrophobicity in transmembrane domains.

Considering all available knowledge, it is clear that any accurate atomistic models considering physics principles relating a sequence to its expression and plasma membrane localization levels aren’t available due to advanced level stochasticity and large scale complexity of the biological process. Statistical models, offering an alternative, are useful to predict the outcomes of any complex processes, because they do rely on energetics of the system (e.g., for ion channels see ref. [17,18]) and not require any prior knowledge of underlying mechanisms. Empirical data such as expression or localization values of MPs’ known sequences can be used to train statistical models. While training, this model infers input/output relationships between the sequence as input and expression or localization as output. These relationships are then used to make predictions on the properties of the unmeasured sequence variants. The process of the use of empirical data for training and selecting optimal statistical models is referred to as ML [14].

In predicting protein properties such as solubility [74], crystallization propensity, periplasm trafficking [75], and general functions [76] ML has been found useful. Although these models can identify protein sequence elements that are predicted to contribute to specific property of interest in the respective studies, they are not generally useful to identify subtle sequences and features, such as amino acids or their interactions. Specific condition of expression and pinpointed localization for any certain class of any related sequences (e.g., of ChRs) aren’t identifiable with confidence. The common reason behind this is that the ML models utilized here are trained using many protein classes and utilizing large data sets that are composed of literature/published data from various sources having almost no standardization on the versatile experimental conditions, and trained using many protein classes. Bedbrook and colleagues focused on a model building on ChRs, utilizing training data that are collected from a considerable range of ChR sequences and under standardized conditions [14]. Here the Gaussian process (GP) classification has been applied and the regression followed from ref. [77] to build ML models that are able to predict expression and localization of ChR directly from the data. Sitting on a background of their earlier work where GP models were found to successfully predict a few biophysical conditions such as the thermal stability, binding affinity, enzyme kinetics, etc. [78]. In this work Arnold group asked whether GP models could successfully predict mammalian expression and heterologous integral membrane localization of ChRs, and to do so the amount of experimental data might be required. To generate a training set, SCHEMA recombination chimeras were used, which are useful for producing large scale libraries of quite diverse, functional chimeric sequences from parent protein homologues [79]. Synthesis and measurements of expression and localization were made for a small subset (0.18%) of the sequences from the recombination library of ChR. These data were used to train GP classification and regression models and predict the expression and localization properties of ChR sequences.

**Figure 14:**
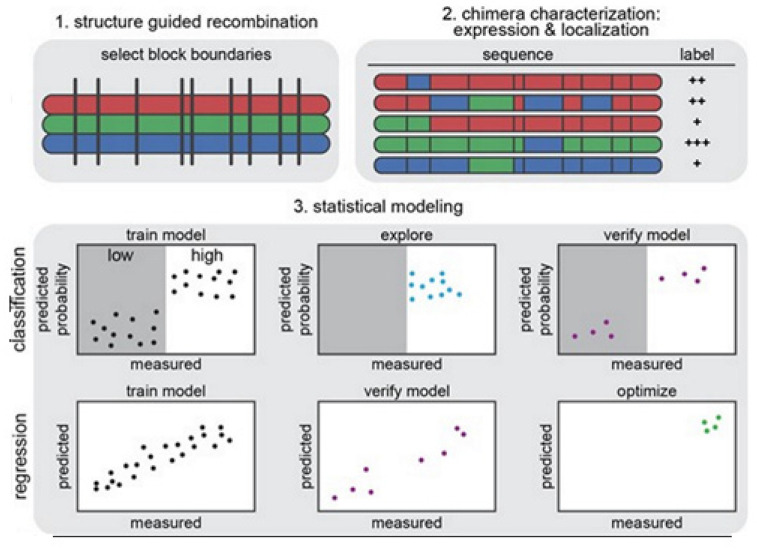
Generalized approaches to the ML of the protein (ChR) structure-function relationships. Here the diversity generation, the measurements on a set for training, and the modeling have been demonstrated.

The strategy on development of the predictive ML models is illustrated in Figure 14 [14]. In Figure 14 (1) we see that the structure-guided recombination SCHEMA is for selecting the block boundaries. This is done for the purpose of shuffling the sequences of the protein and generate any library for sequence-diverse ChRs by starting with 3 ChRs parents, referring to 3 colors (red, green, and blue). In Figure 14 (2), we present a library subset which will serve as the set for the training. The genes of the chimeras have been synthesized and cloned into the mammalian expression vector, transfected cells being assayed for ChR expressions and localizations. In Figure 14 (3), 2 models, classification and regression, are trained with the utilization of the training data and then verified. The model for classification here is used for exploring the diverse sequences that are predicted to show ‘high’ level of localizations. The model for regression here is used for designing the ChRs having the optimal level of localizations to plasma membranes.

**Figure 15:**
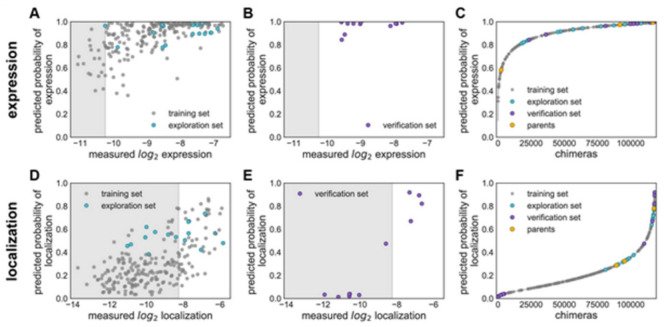
The GP binary classification models for the ChR expression and the ChR localization.

Figure 15 explains various features on building GP classification models of ChR properties, for details including data sets used see ref. [14]. In Figure 15, the plots of the predicted probability versus the measured properties have been divided into many sections, the ‘high’ performer represented by the white background, and the ‘low’ performer represented by the gray background, for each property: expression and localization. In Figure 15A,D, the predicted probability vs. measured properties for the training set (see gray points) and the exploration set (see cyan points). LOO cross-validation was utilized for predictions for training and exploration sets. In Figure 15B,E, the predicted probabilities vs. measured properties for the verification set are presented. A model trained on the training and exploration sets was utilized for predictions. In Figure 15C,F, the predicted probability of the ‘high’ expression, and the localization for all chimeras in the recombination library (having 118,098 chimeras) that is made by the use of the models, trained on data taken from the training and the exploration sets. All the library contained chimeras are shown by the gray lines; the gray, cyan, purple, and yellow points indicating the sets for training, exploration, verification, and the parents, respectively. Figure 15A–C Show the expression and D–F the localization.

**Figure 16:**
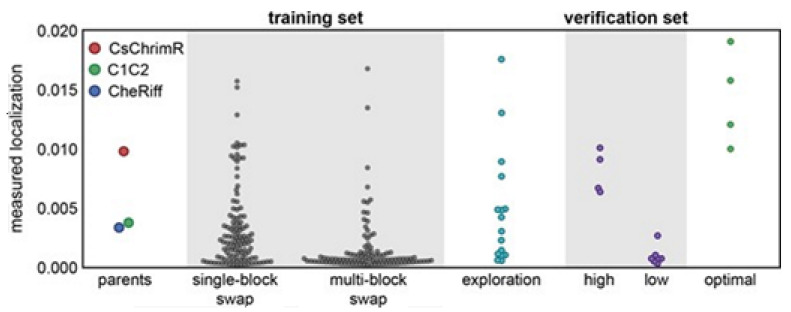
The comparison among all of the measured localizations in the membrane for every set of data. The swarm plots for the measurements of the localizations for each data set compared to the parents: the set of the training, the exploration, the verification, and the optimization.

Multi-block-swap sequences (from the training set) mostly did not localize to the membrane. The model for the localization classification was used for identifying the multi-block-swap chimeras of the library, having a pretty high probability of prediction, >0.4, falling into a ‘high’ localizer category (see previous Figure 15D). Among the multi-block-swap chimeras having the predicted ‘high’ localization, a 16 diverse chimeras set (having average of 69 mutations in amino acids) of the closest parent have been chosen and then named as ‘exploration’, see ref. [14]. Bedbrook and colleagues synthesized and tested these chimeras, and found that the model had accurately predicted chimeras showing good localization (Figure 15 and Figure 16). 50% of the exploration set was found to show ‘high’ localization compared to just 12% of the multi-block-swap sequences from the original training set, although both have similar levels of mutation, data shown in [14]. Exploration set chimeras to have on average 69 ± 12 amino acid mutations from the closest parent versus 73 ± 21 for multi-block-swap chimeras in the training set.

**Figure 17:**
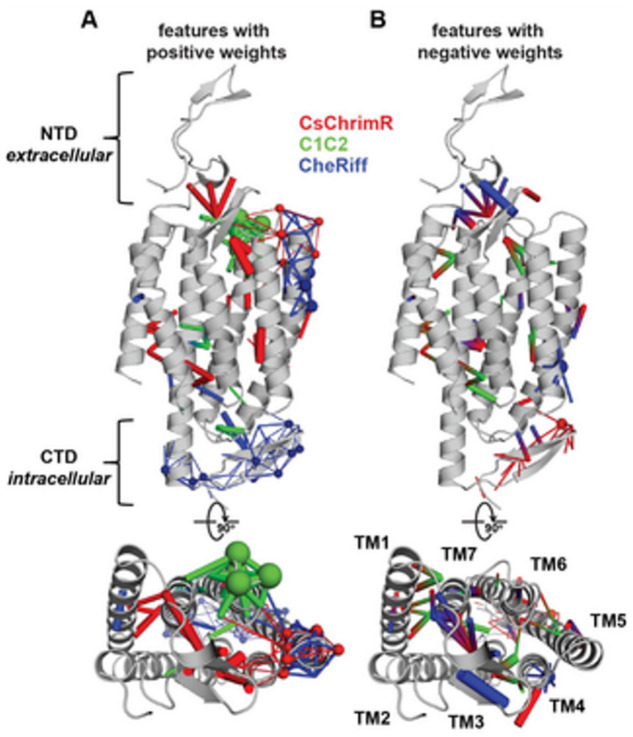
Sequencial and structuralal contact features are important for the prediction of the ChR localizations.

Although the model for the classification predicts the probability of any sequence that falls into the ‘high’ localizer category, it may lack in giving a necessary quantitative prediction. The designing of the chimera sequences having the optimal localization has then been made [14]. For optimal localization, one has to be at or above the CsChrimR level, which is the best localizing parent [80]. A regression model for the localization of ChR in the plasma membrane is required to help predict sequences with optimal localization. A GP regression model is presented which utilized the localization data from training and exploration sets [14]. While developing the GP regression model for the localization, L1-regularized linear regression was used to identify a limited set of sequences and specific structural features that are known to strongly influence the ChR localization. The features include inter-residue contacts and individual residues, and help to offer insight into the structural determinants for the ChR localization. While mapping onto the C1C2 structure, these features can highlight parts of the ChR sequence and the structural contacts which are important for ChR’s plasma membrane localization, see Figure 17 [14]. Both beneficial and deleterious features are distributed throughout the protein, with no single feature dictating localization properties (Figure 17).

In Figure 17, the features with the positive (A) and the negative (B) weights have been displayed on the crystal structure of C1C2 (in grey color). The features can be the residues (see spheres) or the contacts (sticks) from the ChRs parents. The CsChrimR features have been shown in color ‘red’, the features from the C1C2 are presented in color ‘green’, and the features from the CheRiff are presented in color ‘blue’. For cases where any feature appears in 2 parents, the color priorities have been used differently, as follows: the red above and the green above the blue. Sticks are shown to connect beta carbons of the contacting residues (or specifically the alpha carbon for the glycine). The spheres’ size and the sticks’ thickness have been used as proportional to the weights of the parameter. 2 contact residues can either be from the same or different parents. The Single-color contacts occur as both contributing residues appear from the same parent. The occurrence of multi-color contacts happens when residues from different parents come in contact. N-terminal domain (NTD), C-terminal domain (CTD), seven transmembrane helices (TM1-7) are labeled.

**Figure 18:**
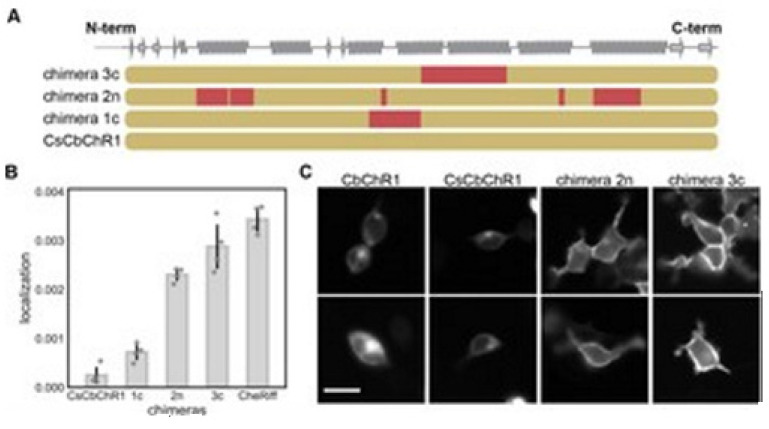
GP regression model that enables the engineering of the localization in CbChR1.

GP regression model as briefed here can be utilized to engineer novel sequences that localize better [14]. Bedbrook and colleagues chose a nonfunctional natural ChR variant, CbChR1, expressed in HEK cells and neurons, but does not localize to the plasma membrane [80]. Being distant from three parental sequences CbChR1 is only 60% identical to CsChrimR and 40% to CheRiff and C1C2. CbChR1 was optimized by introducing minor changes in amino acids, predicted by the localization regression model might be beneficial for membrane localization. To enable CbChR1 localization measurements with the SpyTag-based labeling method, the N-terminus of CbChR1 was substituted with the CsChrimR N-terminus that contains the SpyTag sequence downstream of the signal peptide to make the chimera CsCbChR1 [81]. This block swap was not found to cause any change in CbChR1′s membrane localization properties, see Figure 18C.

Figure 18A presents the blocks’ identities in CsCbChR1 chimeras-each row is representing a chimera. Yellow color representation for the CbChR1 parent and red color representation for the CsChrimR parent. The Chimeras 1c, 2n, and 3c contain 4, 21, and 17 mutations, respectively with respect to the CsCbChR1. In Figure 18B, the plot represents the measured CsCbChR1 localization, compared to 3 CsCbChR1 single-block-swap chimeras and CheRiff parent. In Figure 18C, 2 cell images of mKate expression in CbChR1 and CsCbChR1 compared with top-performing CsCbChR1 single-block-swap chimeras showing the differences in the ChR localization properties–the chimera 2n and the chimera 3c localize to the plasma membrane. The bar of the scale here is 20 μm.

The classification and the models of the regression models of the GP were trained using the expression and the localization data that were collected from the 218 ChR chimeras, which were chosen from the library of 118,098 variants designed using the SCHEMA recombination of three ChRs parents. The GP models were used for identifying the ChRs, which were expressed and well-localized, showing that these models elucidate the sequence and the structure elements important for the processes.

Bedbrook and colleagues have successfully detailed the steps in building ML models and highlighted these artificial techniques’ power in predicting certain protein properties considering a specific ion channel protein ChR [14]. Combining recombination-based library design with statistical modeling methods, they could scan a highly functional portion of protein sequence space through training on only a few sequences. Model developments have yielded a tool having been used to not only predict optimally performing chimeric proteins but also be applied to improve related ChR proteins that are outside the library. These ML methods may appear as powerful tools for general protein engineering in general. As shown here for ChR these ML models may also be found applicable to regulate any ion channel functions by engineering the channel proteins.

## 8. Conclusions

The understanding of the ion channels has historically been made using mostly biological, biochemical, and biophysical principles and techniques. Both bioinformatics and genomics of ion channels have recently appeared as important areas of research that often attract the applications of artificial intelligence techniques including machine learning and deep learning models, and algorithms. The easy analysis of huge amounts of data explaining various ion channel features, such as channels’ structures, functions, classification, channel subunit protein evolution, mutations, etc. has thus been found possible. Application of artificial intelligence techniques in biological systems [82,83], e.g., ion channels, requires the development of specific algorithms and models capable of connecting with the complex, dynamic, and fluctuating natures of biomolecules involved in channel structures. An in-depth analysis in this regard has been provided. This review article has made important guidelines that will hopefully help the ion channel research scientists working towards further developments. Thus the article may be considered an unavoidable reference for subsequent studies.

## Figures and Tables

**Table 1:** Ion Channel modeling and simulation studies. The references quoted in the table are readily found as referenced in article [24]. Here the general area of ion channels are organized according to the system type and computational models employed. “Reprinted (adapted) with permission from [24]”.

System	Method (We Quote Here the References, Numbered in ref. [24]. We Avoided to Relist the Huge Amount of References Here.)					
	Continuum	Implicit Solvent MD	All-Atom MD	Hybrid	CG	Others (QM)
gramcidins	8–15	16, 17	18–31	52, 53		54
Other membrane porins	55–61	55, 62–64	55, 65–87	55		
α-hemolysin	88–93	88, 90, 94, 95	90, 93, 96–99	90, 93, 100–102		
K^+^ channels	103–109	110–117	29, 88, 111–113, 116–197	107, 198–202	203–206	207–211
nAChR			212–220			
MscL/MscS	221–228	229, 225	230–257	225, 258	222, 258	
Anion channels (VDAC, CIC)	259		260–264	265–268		
Aquaporins			269–274			
NH_4_^+ transporter^			275–278			
Other channels	279–310	311–318	299, 312, 319–348	302, 330, 337, 349–356	357, 358	
Synthetic nanopores	359–370	371–374	375–391	350, 382, 383, 392	393	394

**Table 2:** Ion channel families [15].

Abbreviation	Full Names	Function
Ano	Anoctamin, Ca^2+^ activated Cl^−^	Smooth muscle, excitability
ASC	Epithelial (ENaC), acid sensing channel (ASIC)	Osmoregulation, synaptic transmission
CNG/HCN	Cyc. nucleotide gated	Sensory transduction, heart
Ca_v_	Voltage-gated Ca^+^ channel	AP, muscle contraction, secretion
ClC	Voltage-gated Cl^−^ channel	Muscle membrane potential, kidney
GIC	Glutamate receptor (iGluR)	Synaptic transmission
LIC	Ligand-gated, Cys-loop receptor	Synaptic transmission
K_v_	Voltage-gated K^+^ channel	AP, membrane potential regulation
Na_v_	Voltage-gated Na^+^ channel	AP propagation
Leak	Sodium leak (NALCN), yeast calcium channel (Cch1)	Regulation of excitability
P2X	Purinurgic receptor	Vascular tone, swelling
PCC	Polycystine, Mucolipin	Sensory transduction, kidney
RyR	Ryanodine receptor, IP_3_ receptor	Intracellular, muscle contraction
Slo	Voltage and ligand-gated K^+^	AP, resting potential
TPC	Two-pore channel	Intracellular, NAADP signaling
TRP	Transient receptor potential	Sensory transduction

**Table 3:** Prediction results of the ion channels and the non-ion channels.

Method	Ion Channel (%)	Non-Ion Channel (%)	OA (%)
Random forest (188D)	90.3	77.2	83.7793
SVM (188D)	87.0	78.5	82.7759
Random forest (400D)	87.7	77.5	82.6087
SVM (400D)	86.6	83.7	85.1171
Random forest (588D)	77.5	90	83.7793
SVM (588D)	83.2	80	81.6054
Random forest (587D)	77.2	89.7	83.4448
SVM (587D)	77.2	83.3	80.2676

**Table 4:** Compare the results of the voltage-gated ion channels with that of the ligand-gated ion channels.

Method	Voltage-Gated Ion Channel (%)	Ligand-Gated Ion Channel (%)	OA (%)
Random forest (188D)	93.9	86.0	89.9329
SVM (188D)	91.9	86.7	89.2617
Random forest (400D)	88.5	82.7	85.5705
SVM (400D)	82.4	83.3	82.8859
Random forest (588D)	89.2	86.0	87.5839
SVM (588D)	91.9	86.7	89.2617
Random forest (188D)	92.6	86.7	89.5973
SVM (188D)	91.9	86.7	89.2617

**Table 5:** Prediction results for the voltage-gated ion channels-four types.

Method	K (%)	Ca (%)	Na (%)	Anion (%)	OA (%)	AA (%)
Random forest (188D)	97.5	37.9	50	46.2	72.973	57.9
SVM (188D)	96.3	48.3	58.3	69.2	79.0541	68.0
Random forest (400D)	97.5	6.9	50	23.1	62.8378	44.4
SVM (400D)	85.2	62.1	50	73.1	75.6757	67.6
Random forest (588D)	97.5	34.5	50	57.7	74.3243	59.9
SVM (588D)	96.3	48.3	58.3	69.2	79.0541	60.2
Random forest (424D)	98.8	34.5	58.3	46.2	73.6486	59.5
SVM (424D)	96.3	48.3	58.3	69.2	79.0541	68.0

**Table 6:** SNPs in 237 ion channel genes in subjects having idiopathic generalized epilepsy and neurologically normal individuals.

Type/Location of SNP	Number of Validated SNPs ^1^	Percent of Validated Dataset (%)	Number of Novel SNPs Discovered	Number of Validated SNPs per Megabases Sequenced ^6^		
				Cases Only (n = 152)	Controls Only (n = 139)	SNPs in Both Cases and Controls (n = 291)
Promoter ^2^	80	2.6	18	0.4	0.1	0.4
5′ UTR	79	2.6	7	0.2	0.1	0.5
3′ UTR	461	14.9	62	1.4	0.6	3.0
Synonymous (sSNP)	936	30.2	351	5.1	2.2	4.2
Nonsynonymous (nsSNP)	668	21.6	415	4.9	2.2	1.9
Nonsense/Stop Codon	9	<1	9	0.1	0.03	0
Splice Site SNP ^3^	12	<1	9	0.1	0.03	0.02
Splice Region SNP ^4^	90	2.9	13	0.3	0.1	0.6
Intron SNP	737	23.8	101	2.3	1.0	4.7
Undefined ^5^	23	<1	4	0.1	0	0.2
TOTALS	3095	100.0	989	14.6	6.3	15.6

^1^ validated SNPs combining 1. visual validation, 2. previous discovery (dbSNP ID), 3. detected on a custom MIP chip, 4. Biotage and/or 454 sequencing. ^2^ SNPs in promoter regions. ^3^ splice site (+2 to −2 bp in exon boundary at splice junction). ^4^ splice region (−2 to −15 bp in exon boundary). ^5^ undefined SNPs. ^6^ number of individual SNPs per megabase sequenced.

**Table 7:** Tissue Info expression screen ^1^.

Chromosome	Band	Ensembl Transcript ID	Hit(s)	Hit(s) in Hippocampus ^2^	Tissue Summary	Gene Name/Other ID
1	p34.3	ENST00000319637	2	2	hippocampus	EPHA10
2	p21	ENST00000306078	2	1	hippocampus	KCNG3
2	q37.1	ENST00000313064	2	1	hippocampus	C2orf52
6	q15	ENST00000303726	3	1	hippocampus	CNR1
6	q25.3	ENST00000308254	1	1	hippocampus	Retired in Ensembl 46
6	q27	ENST00000322583	1	1	hippocampus	NP_787118.2 (Link)
9	q21.33	ENST00000298743	3	1	hippocampus	GAS1
10	q24.33	ENST00000329905	3	2	hippocampus	CALHM1 (FAM26C)
11	q24.1	ENST00000354597	3	1	hippocampus	OR8B3
17	q25.3	ENST00000326931	2	1	hippocampus	Q8N8L1_HUMAN
19	p12	ENST00000360885	1	1	hippocampus	Retired in Ensembl 46
X	q27.2	ENST00000298296	1	1	hippocampus	MAGEC3

^1^ One transcript is shown for each gene identified in the screen. Genomic location and number of hit(s) in dbEST are reported for each transcript. ^2^ Hit(s) in hippocampus indicates how many ESTs matching the transcript were sequenced from a cDNA library made from the hippocampus. Link: https://www.ncbi.nlm.nih.gov/protein/NP_787118.2 (26 June 2021).

**Table 8:** Allele and genotype distributions of the CALHM1 P86L polymorphism (rs2986017) in AD case and control populations.

		Allele Distribution (%)		Genotype Distribution (%)		
	**n**	**C**	**T**	**CC**	**CT**	**TT**
USA screening sample ^1,2^						
Controls	23	36 (0.78)	10 (0.22)	14 (0.61)	8 (0.35)	1 (0.04)
Autopsied AD cases	46	59 (0.64)	33 (0.36)	20 (0.44)	19 (0.40)	7 (0.16)
**France I** ^3,4^						
Controls	565	907 (0.80)	223 (0.20)	370 (0.65)	167 (0.30)	28 (0.05)
AD cases	710	1051 (0.74)	369 (0.26)	410 (0.58)	231 (0.32)	69 (0.10)
**France II** ^5,6^						
Controls	483	716 (0.74)	250 (0.26)	271 (0.56)	174 (0.36)	38 (0.08)
AD cases	645	888 (0.69)	402 (0.31)	303 (0.47)	282 (0.44)	60 (0.09)
**UK** ^7,8^						
Controls	205	320 (0.78)	90 (0.22)	127 (0.62)	66 (0.32)	12 (0.06)
AD cases	365	504 (0.69)	226 (0.31)	193 (0.53)	118 (0.32)	54 (0.15)
Autopsied AD cases	127	169 (0.66)	85 (0.34)	57 (0.45)	55 (0.43)	15 (0.12)
**Italy** ^9,10^						
Controls	85	131 (0.77)	39 (0.23)	52 (0.61)	27 (0.32)	6 (0.07)
AD cases	150	210 (0.70)	90 (0.30)	74 (0.49)	62 (0.41)	14 (0.09)
**Combined studies** ^11,12^						
Controls	1361	2110 (0.77)	612 (0.23)	834 (0.61)	442 (0.32)	85 (0.06)
AD cases	2043	2881 (0.71)	1205 (0.29)	1057 (0.52)	767 (0.37)	219 (0.11)

^1^*p* = 0.10; ^2^ *p* = ns; ^3^ *p* = 0.0002; ^4^ *p* = 0.001; ^5^ *p* = 0.006; ^6^ *p* = 0.01; ^7^ *p* = 0.0002; ^8^ *p* = 0.00002; ^9^ *p* = 0.10; ^10^ *p* = ns; ^11^ *p* = 2 × 10^−10^; ^12^ *p* = 7 × 10^−^9; OR (CT vs. CC) = 1.37, 95% CI [1.18–1.59], *p* = 3 × 10^−5^; OR (CT vs. CC) = 1.27, 95% CI [1.08–1.50], *p* = 0.004 adjusted for age, gender, APOE status, and center; OR (TT vs. CC) = 2.03, 95% CI [1.56–2.65], *p* = 2 × 10^−7^; OR (TT vs. CC) = 1.77, 95% CI [1.33–2.36], *p* = 9 × 10^−5^ adjusted for age, gender, APOE status, and center; ns, non-significant.

**Table 9:** The data sets used in this experiment. Due to copyright issues, the table is reconstructed using data from ref. [69].

Sets of Data	The Original Data	Similarity, Less than 20%	Testing Data	Training Data
Ion channel	845	301	60	241
Ion transporter	1051	351	70	281
Membrane protein	8295	4263	850	3413
Total	10,191	4915	980	3935

**Table 10:** Comparison of the performance on the classification of the ion transporters and the ion channels from a set of the membrane proteins utilizing 5-fold techniques of the cross validation. Due to copyright issues, the table is reconstructed using data from ref. [69].

Data Sets	Sen	Spec	Acc	MCC
Ion channels (class A)	89.20	84.89	87.05	0.75
Ion transporters (class B)	86.76	88.23	87.49	0.75
Other proteins (class C)	92.50	96.19	94.35	0.89

## Data Availability

Not applicable.
